# Non-uniform radiation-induced biological responses at the tissue level involved in the health risk of environmental radiation: a radiobiological hypothesis

**DOI:** 10.1186/s12940-018-0444-4

**Published:** 2018-12-29

**Authors:** Hisanori Fukunaga, Kevin M. Prise

**Affiliations:** Centre for Cancer Research and Cell Biology, Queen’s University Belfast, 97 Lisburn Road, Belfast, BT9 7AE UK

**Keywords:** Environmental radiation, Radiation-induced biological effects, Tissue sparing effects, Health risk assessment, Radiological protection

## Abstract

**Background:**

The conventional concept of radiation protection is based on epidemiological studies of radiation that support a positive correlation between dose and response. However, there is a remarkable difference in biological responses at the tissue level, depending on whether radiation is delivered as a uniform or non-uniform spatiotemporal distribution due to tissue sparing effects (TSE). From the point of view of radiation micro-dosimetry, environmental radiation is delivered as a non-uniform distribution, and radiation-induced biological responses at the tissue level, such as TSE, would be implicated in individual risk following exposure to environmental radiation.

**Hypothesis:**

We hypothesize that the health risks of non-uniform radiation exposure are lower than the same dose at a uniform exposure, due to TSE following irradiation. Testing the hypothesis requires both radiobiological studies using high-precision microbeams and the epidemiological data of environmental radiation-induced effects. The implications of the hypothesis will lead to more personalized approaches in the field of environmental radiation protection.

**Conclusion:**

The detection of spatiotemporal dose distribution could be of scientific importance for more accurate individual risk assessment of exposure to environmental radiation. Further radiobiological studies on non-uniform radiation-induced biological responses at the tissue level are expected.

## Background

Radiation-induced effects on biological tissues were recognized immediately after the pivotal discovery of X-rays by Wilhelm Röntgen in 1895 [1]. The first radiation-related solid cancer was reported in 1902, arising in an ulcerated area of the skin, and, as reported in 1911, leukemia was diagnosed in five radiation workers [2]. According to the results of previous epidemiological studies, including the Life Span Study (LSS) of Japanese atomic bomb survivors, these biological effects seem to be dose-dependent with or without a threshold [3–6], thus the current concept of radiological protection is based on the dose-response model. In fact, for more than four decades, according to reports and statements of radiation research organizations, such as the US National Council on Radiation Protection and Measurements (NCRP) and the International Commission on Radiological Protection (ICRP), a linear non-threshold (LNT) model has been used for radiological protection purposes [7, 8]. In May 2018, NCRP published the Commentary No. 27, which supports the LNT model for radiological protection, based on recent results of epidemiological studies of radiation [9], although there are technical limitations to epidemiology, such as study design, sample size and confounding factors. Thus, the risk assessment of environmental radiation exposure is still based on the LNT model, although quantifying the risk still remains problematic and subject to uncertainty [10].

From the perspective of recent radiobiology, we highlight a specific challenge: the contribution of spatiotemporal dose distribution might be underestimated in the field of radiological protection. In an epidemiological cohort study of radiation, the researchers collected the data of the total radiation dose each participant was exposed to; however, it is technically difficult to have a clear grasp of their accurate irradiation situations, such as radiation dose rates or spatial distributions in the whole body. This is one of the possible technical limitations of the current epidemiological approach to radiation.

**Fig. 1:**
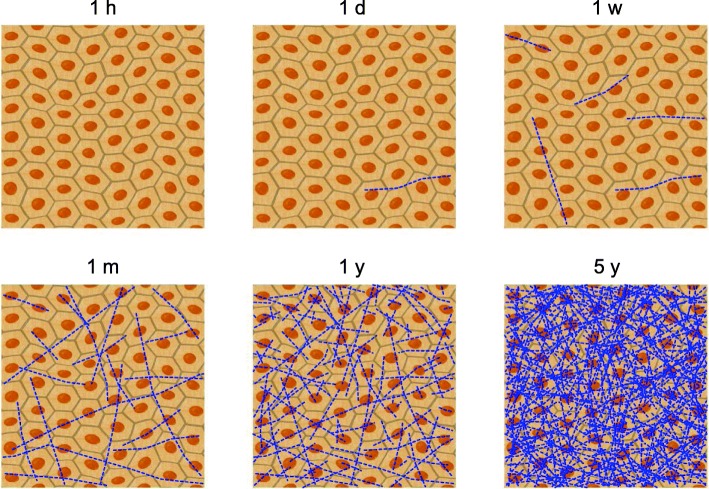
Low-dose radiation dose distributions at micro-scale level. This is a simulated result of exposure to dose levels of approximately 1 mGy/year. The distribution of the dose is tempo-spatially heterogenous. The blue lines indicate radiation tracks in the irradiated tissue

The quantity and quality of DNA damage is determined by the radiation type and dose [11]. For instance, high and low linear-energy-transfer (LET) radiations induce different spectra and qualities/complexity of DNA lesions, due to the differences in radiation track structures [12]. This also impacts on the dose per cell delivered by individual tracks at low doses which will be radiation quality dependent. Specifically, as shown in Fig. 1, from the point of view of radiation micro-dosimetry, for low-dose exposure, such as from environmental radiation, or low-dose rates of radiation, the energy deposition of radiation is localized along its track, resulting in a non-uniform distribution of exposed or unexposed cells in irradiated tissue [13, 14]. Thus, for environmental radiation, there are possible interactions between the irradiated and the non-irradiated cells and the dynamics of these cells in the tissue involved in radiation-induced biological responses at the whole-tissue level [15]. In fact, it is well known that cells that do not receive radiation doses directly but receive signals from nearby or neighboring irradiated cells behave as though they have been exposed [16, 17]. These well-documented responses are collectively known as non-targeted effects, although the underlying molecular mechanisms are not completely understood [18]. However, the conventional concept of radiological protection has not taken into consideration such tissue-level responses following exposure to environmental radiation.

In clinical practice, the tissue-sparing response in non-uniform radiation fields was recognized more than one century ago. In 1909, Alban Köhler reported the first clinical observation of a tissue-sparing response during grid radiotherapy in which spatially fractionated radiation was delivered using a grid-like pattern of beams [19]. In 1995, a notable tissue-sparing was reported in rat brain tissues during a microbeam radiation therapy (MRT) study [20], performed at the National Synchrotron Light Source, Brookhaven National Laboratory. Since then, the tissue-sparing effect (TSE) of MRT, which is based on a spatial fractionation of synchrotron-generated X-ray microbeams at the microscale level, has been confirmed in a large variety of species and tissue types, although the underlying mechanism of TSE remains to be established [19–25]. The TSE of spatial-fractionated radiation indicates significant implications not only for clinical applications, but also for the improvement of risk assessment of exposure to non-uniform radiation, such as environmental radiation. For a more accurate risk assessment of exposure to environmental radiation, the assessment of the spatiotemporal dose distribution could be of scientific importance due to the TSE.

### Presentation of the hypothesis

**Fig. 2:**
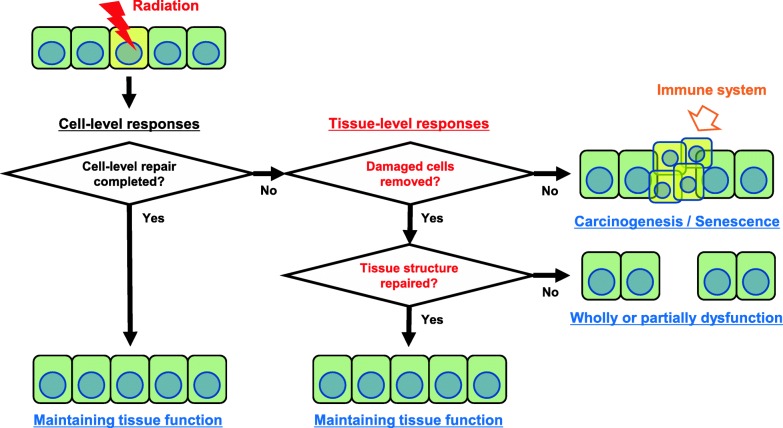
Radiation-induced biological responses at the tissue level. When cell-level repair responses (e.g. DNA damage repair, and oxidative stress response) cannot completely repair the radiation-induced damages, the removal of damaged cells (e.g. apoptosis, and cell competition) and tissue structure repair/regeneration (e.g. stem cell migration and proliferation) minimize the influence for maintaining normal tissue functions. The removals of damaged cells prevent the tissue from carcinogenesis or senescence that are targets of immune system. The failures of tissue structure repair/regeneration induce wholly or partially the dysfunction

As shown in Fig. 2, intercellular responses, such as apoptosis and clearance of apoptotic cells, cell competition and tissue repair/regeneration, would be involved in TSE in response to non-uniform radiation fields. Depending on the efficiency of acute cellular responses following irradiation, the damaged cells either survive or are removed from the tissue. In general, if radiation-induced damages of cellular components, in particular DNA, are not repaired sufficiently, the damaged cells will commit suicide in a process called apoptosis, which is a clearance system in multicellular organisms [26, 27]. Further, for tissue homeostasis, cell competition is essential as a cell fitness-sensing mechanism seen from insects to mammals that eliminates cells that, although viable, are less fit than their neighbors [28]. Damaged cells induced by non-uniform radiation would be removed by the neighboring cells due to cell competition, resulting in the prevention of a pathological state, such as carcinogenesis [29]. In addition, to remove cancer cells, interactions between the immune system and cancer governed by a complex network of biological pathways are a rapidly developing research area [30]. The therapeutic effects of radiotherapy have been observed not only in cancer cells, but also in their microenvironment. Today the role of the host’s immune system in the mechanisms of tumor regression by generating a cytotoxic adaptive immune response is well described and is recognized as immunogenic cell death (ICD) [31]. In this complex myriad of events, intrinsic characteristics of the tumor cells (tumor type, immunogenic capacity) and the immune status of the host are important factors determining the successful induction of ICD [32]. In addition to the potential synergism in terms of local control, the possibility to obtain systemic responses, described initially by Mole in 1953 as “the abscopal effect [33],” mediated by ICD has stimulated great interest. After the complete clearance of damaged cells, tissue repair/regeneration generally occurs for the maintenance of normal tissue functions, namely homeostasis. Somatic stem cells migrate from the intact to the defective parts and regenerate the structure and function of tissue by their proliferation and differentiation [34, 35]. Such tissue homeostasis mechanisms could be involved in radiation-induced biological responses at the tissue level. However little is know, particularly with respect to immune system modulation at low environmental doses.

We do not intend to question the conventional LNT concept for radiological protection purposes. Our aim is to suggest further studies on the mechanisms of radiation-induced biological responses at the tissue level for a more accurate estimation of environmental radiation risk. We hypothesize that the health risks of environmental radiation exposure are prone to be overestimated because at the very least radiation-induced biological responses at the tissue level, especially TSE, are not adequately considered. Conventionally, animal studies have been commonly used to investigate the radiation-induced biological responses at the tissue level. However, most of these studies have been performed by uniform radiation exposure, such as total body irradiation, thus the knowledge on tissue homeostasis responses following exposure to non-uniform radiation, such as environmental radiation, appears to be insufficient even now.

### Testing the hypothesis

Because the technology of MRT has only recently been developed, the molecular mechanisms of TSE in response to non-uniform radiation fields have not yet been fully determined. As we suggest in this paper, there appears to be several potential biological responses involved in tissue homeostasis after exposure to non-uniform radiation, such as environmental radiation. Given the use of high-precision microbeams, further radiobiological studies on such biological responses are foreseen. Key to this will be an appropriate assessment of the interrelationship between dose and its localization in tissue volumes.

Also, epidemiological studies of non-uniform radiation-induced biological effects will provide a more comprehensive radiological understanding of response mechanisms, leading to improved accuracy in the estimations of environmental radiation risk. The confirmation of spatiotemporal dose distribution of environmental radiation will be of scientific importance in such studies.

### Implications of the hypothesis

The hypothesis will suggest reconsidering the concept of the health-risk assessment of environmental radiation from the viewpoint of precision medicine. If the evidence for the hypothesis can be strengthened by appropriate radiobiological and epidemiological studies, the target of current environmental radiation risk assessment approaches will show a dramatic change from the “average person” to “each person”. To consider individual spatiotemporal exposure to radiation will provide a novel insight into individual risk assessment of environmental radiation. For the coming era of precision medicine, in addition to the consideration of genomic factors [36], the global scientific community, including such bodies as NCRP and ICRP, need to consider how to integrate similar personalized approaches into a future concepts of radiological protection. We hope that our hypothesis will stimulate scientific debate in the field.

Since the large-scale nuclear disasters in Chernobyl, in 1986, and in Fukushima, in 2011, there has been a great deal of public concern about the possible health effects of long-term and low-dose radiation exposure on current and future generations [37–39]. Previous data based on population cohorts, such as the LSS, estimate that the risk of cancer from radiation exposure will increase at doses exceeding approximately 100 mSv [4, 5], although there are technical limitations and biases [40]. These data, however, are mainly based on acute irradiation situations, such as the explosion of the atomic bomb, and could not take weighting factors of spatiotemporal dose distributions into consideration. The hypothesis we suggest would provide a novel approach for more accurate individual risk assessment of such low-dose and long-term environmental radiation exposures.

Risk assessment of exposure to environmental radiation is essential for human activity in space. As future missions explore beyond *low-Earth orbit* (LEO) and away from the protection of the Earth’s magnetic shielding, the nature of radiation exposure that astronauts encounter will include higher radiation exposures [41]. During transit outside of LEO, every cell nucleus within an astronaut’s body would be traversed by a proton or electron ray every few days, and by a heavier galactic cosmic ray ion (e.g., O, Si, Fe) every few months [42]. For a more accurate risk assessment of exposure to such an environment, the understanding of non-uniform, radiation-induced biological responses at the tissue level will be of scientific importance.

## Abbreviations

ICD: Immunogenic cell death
ICRP: International Commission on Radiological Protection
LEO: Low-Earth orbit
LET: Linear-energy-transfer
LNT: Liner non-threshold
LSS: Life Span Study of Japanese atomic bomb survivors
MRT: Microbeam radiotherapy
NCRP: National Council on Radiation Protection and Measurements, USA
TSE: Tissue-sparing effects
